# Processing Sentences with Literal versus Figurative Use of Verbs: An ERP Study with Children with Language Impairments, Nonverbal Impairments, and Typical Development

**DOI:** 10.1155/2015/475271

**Published:** 2015-07-12

**Authors:** Maria Luisa Lorusso, Michele Burigo, Virginia Borsa, Massimo Molteni

**Affiliations:** ^1^Scientific Institute IRCCS “E. Medea”, Via Don Luigi Monza 20, 23842 Bosisio Parini, Italy; ^2^San Raffaele University and San Raffaele Scientific Institute, Via Olgettina 60, 20132 Milan, Italy

## Abstract

Forty native Italian children (age 6–15) performed a sentence plausibility judgment task. ERP recordings were available for 12 children with specific language impairment (SLI), 11 children with nonverbal learning disabilities (NVLD), and 13 control children. Participants listened to verb-object combinations and judged them as acceptable or unacceptable. Stimuli belonged to four conditions, where concreteness and congruency were manipulated. All groups made more errors responding to abstract and to congruent sentences. Moreover, SLI participants performed worse than NVLD participants with abstract sentences. ERPs were analyzed in the time window 300–500 ms. SLI children show atypical, reversed effects of concreteness and congruence as compared to control and NVLD children, respectively. The results suggest that linguistic impairments disrupt abstract language processing more than visual-motor impairments. Moreover, ROI and SPM analyses of ERPs point to a predominant involvement of the left rather than the right hemisphere in the comprehension of figurative expressions.

## 1. Introduction

According to language-based theories, the acquisition of abstract concepts is based on the implicit extraction of statistical properties and regularities from input: the frequency of cooccurrence and the interaction of an unknown word with the other words in a linguistic context are fundamental to learn its meaning (e.g., [1–3]). By contrast, according to the embodiment theory, concepts are considered as sets of situated representations, relating to specific meanings according to the context's characteristics or to the activity (i.e., agents, objects, events, and introspective states) in which the concept is used [4–6]. In addition, Lakoff, based on evidence of the activation of the motor and premotor cortex not only during an action observation but also during an action imagination task, claimed that concepts are mapped into the sensorimotor system as perceptions and actions and that the process of conceptualization could be considered as a mental simulation originating from the functional areas devoted to action and perception [7, 8]. The link between concrete and abstract concepts is the conceptual metaphor, which makes nonperceivable entities thinkable through reference to a concrete entity. For instance, the sentences “I picked up a flower” and “I picked up an idea” activate the sensorimotor scheme of “picking up” and the neural areas usually involved in the action [8]. This suggests that the ability to understand metaphoric (in Lakoff's terms) expressions such as “picking up an idea” (or, more generally speaking, nonliteral language) is strictly bound with the ability to represent abstract knowledge, and it may even be considered as an essential component of abstract thinking.

Nonliteral expressions constitute an important part of everyday language, conveying conventional wisdom, social norms, and rules [9]. The ability to understand idiomatic expressions in adolescence correlates with academic achievement [10], while individuals with poor social competence, such as those with schizophrenia, also show difficulties in using this form of communication [11, 12]. Due to their relevance on both the theoretical and the clinical and social level, the mechanisms and processes involved in nonliteral language processing have been extensively studied in the last decades.

Nonliteral language includes idioms, proverbs, metaphors, and ironic speech [9]; these types of language are supposed to be mediated by partially distinct neurocognitive mechanisms [13]. Idioms vary with respect to their literal plausibility, compositionality, and transparency/opacity [14]. These dimensions, along with contextual bias, determine how easily an idiom is acquired and comprehended [13].

The comprehension of figurative language is supposed to involve several cognitive abilities [15], including also Theory of Mind and executive control functions [13]. A debated issue concerns the timing and the priorities that regulate the access to literal and figurative meaning. Traditional views suggest that the search for a figurative meaning begins only after the literal meaning has been rejected on the basis of context information (hierarchical hypothesis) [16]. Alternatively, in “direct access models,” both kinds of meanings would be processed at the same time [17]. The two kinds of mechanisms may represent two extremes of a continuum depending on the degree of lexicalization and conventionality of the metaphor (i.e., the “career of the metaphor” according to Bowdle and Gentner [18]).

Functional neuroimaging studies using PET, fMRI, or EEG tried to identify networks of brain regions involved in the processing of figurative language. Bottini et al. [19] assessed brain activity with PET while participants judged the plausibility of visually presented sentences with either a metaphorical or literal meaning. The processing of literal sentences activated left-hemisphere (LH) regions including the frontal cortex, the temporal areas, the parietal cortex, and the precuneus. The processing of metaphorical sentences additionally recruited the equivalents of Broca's and Wernicke's areas in the right hemisphere (RH). Indeed, the RH seems to be activated each time complex syntactic and/or semantic linguistic structures are processed [19, 20]. Rapp et al. [21] confirmed these results with novel metaphorical and literal sentences carefully matched for syntactic and semantic complexity. In general, behavioral results show that subjects find it more difficult to judge the plausibility of metaphorical rather than literal sentences [19].

The present study addresses a specific instance of figurative expressions, where a verb that is usually combined with concrete nouns is coupled with an abstract noun instead, which induces a figurative interpretation of the action. Verbs may be seen as a “special” grammatical category, in that their status in terms of the abstract-concrete continuum is usually assumed to be on the abstract pole, simply because verbs, different from objects, do not refer to single entities, but rather to the relationship between an agent and an action (intransitive), or an agent, an action, and a patient (transitive verbs) (e.g., [22–24]). However, the representation of the action the verb refers to can be more or less abstract, depending partly on the verb and partly on the arguments that are associated with the verb [22, 25]. Some verbs have the peculiarity to be associated with a very large range of possible arguments; these verbs include “light verbs” or “general purpose verbs” [26, 27] (e.g., “get,” “do,” and “take”); “fictive motion” verbs [28] (e.g., “run” in “the street runs along the river”), where motion verbs are used to describe scenarios where no movement is involved (see [29]); and other types of verbs, such as “break,” “steal,” and “close,” that can be associated with more concrete or more abstract objects (consider, e.g., “close the door” versus “close the deal”) or that, in Lakoff's [8] terms, form the basis of conceptual metaphors (e.g., pick up, throw, and lay down). What is relevant for the present discussion is that the association of such types of verbs with an abstract (compatible) object tends to produce a figurative reading of the verb itself (e.g., “steal one's jewels” versus “steal one's words” or “follow a bike” versus “follow a suggestion”). The figurative use of this type of verbs is a particular instance of figurative language, and even if it is usually not regarded as a “prototypical” type of figurative language in (psycho)linguistic studies (more frequently investigating comprehension of metaphors such as “X-noun is Y-noun,” or “X-noun V-verb”), it is very frequent in language use (see [30]). Based on these considerations, the present study investigated the comprehension of verbs associated with concrete and abstract objects, comparing the performances of children with specific language impairment (SLI) with those of children with nonverbal learning disabilities (NVLD). The study of these clinical populations allows addressing the issue of figurative language processing at both the functional (linguistic versus visual-spatial) and the neuroanatomical level (predominantly LH versus predominantly RH impairment). In fact, children with SLI, as will be better detailed in the following paragraph, suffer from a dysfunction affecting language abilities, presumably due to inefficient activation of left-hemisphere functions [31], whereas children with NVLD show poor performance on visual-spatial tasks, presumably caused by right-hemisphere dysfunctions [32]. In other terms, any difference in the processing of figurative language in the two groups may be interpreted as a consequence of their lacking language versus visual-spatial abilities and of the imbalanced contribution/activation of their LH versus RH in the processing of figurative expressions. Thus, according to language-based theories, children with specific deficits in language development should show greater impairments in abstract language processing as compared to children with NVLD. By contrast, according to theories of embodied cognition, children with a specific deficit in sensory-motor functions (i.e., children with NVLD) are expected to perform worse than children with SLI.

More precisely, specific language impairment is a label given to different clinical situations, which are characterized by a delay or a deficit in one or more areas of language development (i.e., phonological, lexical, morphosyntactic, or pragmatic) in the absence of cognitive, sensory, motor, or emotional problems and of sociocultural deprivation [33, 34]. Although linguistic skills are the most affected in SLI, children with this developmental disorder may also show, compared to typically developing children, a lower level of symbolic play [35, 36] and lower performances in mental rotation tasks [37], in the quality and speed of information processing [38, 39], and in conceptual reasoning, as assessed by Piagetian tasks [40, 41].

The label nonverbal learning disability (NVLD), sometimes also referred to as “right-hemisphere syndrome,” different from SLI, is not yet fully recognized as a nosographic category in international diagnostic manuals, but it has been extensively described and investigated by several researchers in the field of neurodevelopmental disorders (e.g., [42–44]). This disorder is characterized by significant deficits in the sensory-motor system, such as perceptual and tactile problems, bilateral motor coordination deficits, and difficulties in visual-spatial organization and memory. These children also have difficulties in problem solving and in the acquisition of visual-spatial concepts; moreover their ability to establish causal relationships and to esteem the flow of time during daily activities appears to be relatively inadequate. Although the cognitive level of children with NVLD is within the normal range, they present a wide gap between verbal and nonverbal IQs [43]. Social-emotional skills are also often affected in this population [44, 45].

The present study employed both behavioral and electrophysiological measures to investigate comprehension of verb-object combinations. At the behavioral level, the processing of verb-object combinations with concrete and abstract objects was investigated through a paradigm of congruency judgment. In addition, ERPs were recorded during the judgment task. Indeed, ERPs can provide useful information concerning language processing mechanisms, and they can be a more sensitive measure than reaction times [46, 47]. One of the most established language-related ERP components is the N400, a negative component starting around 200–250 ms after stimulus onset and peaking at around 400 ms. N400s can be observed for both visual and auditory words, with auditory N400s tending to begin earlier, last longer, and have a slightly more frontal and less right-biased topography (reviewed in [48]). Moreover, the N400 has been used in the study of many different conditions, including dementia, aphasia, autism, cerebral palsy, closed head injury, dyslexia and other developmental language disabilities, and schizophrenia (see [49, 50]). Last but not least, its involvement in language and memory functions has been described across the lifespan, with studies on both typically and atypically developing children. Atchley et al. [51] described the characteristics of N400 in children as being generally greater in amplitude, more delayed in latency (with both delay and amplitude decreasing with increasing age of the children), and more widely distributed in terms of scalp location than that observed in adults [52].

The N400 was first observed in semantically incongruent sentences [53]. Since then, several studies have addressed the functional meaning of this component. First of all, the precise meaning and nature of N400 in congruency effects have been an object of debate in recent studies. In a review paper, Lau et al. [54] showed that one of the important neurogenerators of N400 lies within the posterior middle temporal cortex, which is thought to serve lexical-semantic processing at the word level. This provides support for the lexical account and discourages an interpretation based purely on integration processes. On the other hand, Brouwer et al. [55] view N400 as strictly lexical and argue that postlexical integration is reflected in the late positive component. Many laboratories demonstrated that semantic anomalies are neither necessary nor sufficient for N400 elicitation (a P600 is sometimes observed instead; see [56]) and that modulations of the N400 do not always correspond to response times (RT) patterns. The amplitude of the N400 response elicited by words in sentential contexts is modulated not only by the degree of anomaly per se, but also by the predictability of the ending itself, regardless of congruence [54].

Most importantly, the N400 has been shown to be modulated by the concrete-abstract dimension. ERPs for concrete words show an N400-like long-lasting negativity, starting between 300 and 500 ms after word onset, compared to ERPs for abstract words [57–61]. Also Tsai and colleagues [62] found that, in a lexical decision task and a semantic relatedness judgment task, concrete nouns elicited larger N400 responses than abstract nouns. In contrast to the centroparietal distribution of the classical N400 effect for written words, the N400 concreteness effect seems to extend to frontal electrodes [63] and to persist beyond the standard N400 time window. Nittono et al. [64] found that ERP components were more left-lateralized for low imagery words than for high imagery words and suggested that right-hemisphere activation probably had to do with imagery-related information, which was not available for low imagery words. However, concreteness effects are not always observed, and behavioral data are not always consistent with ERP responses. For instance, Barber et al. [65] found that lexical decision responses were faster for abstract than for concrete words; nonetheless, the pattern of ERP differences was similar to that reported in previous studies, with larger N400 for concrete words. Since the words in the concrete and abstract condition were matched on several lexical and sublexical variables, including imageability, the differences in ERP responses cannot be attributed to such variables and are hypothesized to depend on a differing degree of activation and integration of multimodal (sensory-motor) features from distributed cortical networks [65], consistent with the context availability theory.

A third line of research has focused more explicitly on the literal-figurative dimension in sentence processing. ERP studies on the comprehension of figurative language [46, 66] or jokes [67] mainly reported N400 effects or combinations of N400 and P600, suggesting that nonliteral language requires effortful semantic processing. The semantic context seems to play a prominent role in these results. Pynte et al. [68] found that both familiar and unfamiliar metaphors elicit larger N400s than literal categorical statements. Moreover, since, regardless of familiarity, contextually appropriate metaphors elicited smaller N400 than contextually inappropriate ones, they concluded proposing a model based on context appropriateness. Altogether, ERP studies on metaphoric language do not provide univocal results. Some of the inconsistency may result from confounding variables concerning the linguistic forms of the metaphors and their degree of conventionality [66], as highlighted by the “career of metaphor” model [69].

ERPs have also been used to provide additional information as to the involvement of the two hemispheres in figurative language processing. Previous electrophysiological experiments [57, 59, 64] suggest that the RH is better at processing concrete rather than abstract words, whereas the LH shows equivalent responses to both. According to these results, processing of abstract concepts is left-lateralized, whereas processing of concrete concepts is bilateral, consistent with the dual-coding theory [70]. According to context availability theory [71], by contrast, abstract and concrete words draw on the same neural substrates, with generally stronger activation elicited by concrete words. Previous studies [58, 60] also suggest that concreteness effects are more evident when words are out of context than when they are placed into supportive contexts. Integrated models would suggest that the LH processes both abstract and concrete words, but it is particularly sensitive to congruency and predictability, whereas the RH specifically contributes to the processing of concrete words, probably through imagery activation, as observed in sustained negativities over frontal areas with latencies extending to 500–700 ms after stimulus onset.

ERP data on clinical populations of children are very sparse. Sabisch et al. [72] compared ERP responses in children with SLI and children with normal language development. Children heard correct sentences and sentences violating the selectional restriction of the verb. Control children showed an N400 followed by a late positivity for the incorrect sentences (as shown also by Hahne et al. [73]). By contrast, children with SLI showed no N400 effect but rather a late, broadly distributed positivity. At the behavioral level, children in both groups were better at identifying semantic violations rather than correct sentences. The processing of figurative language in the present investigation was studied through an ERP paradigm similar to Holcomb et al. [57], where participants were asked to listen to a series of sentences in a word-by-word congruency judgment task. However, while Holcomb et al. used full sentences to manipulate the effect of context, only verb-object pairs were used in the present study. Specifically, verbs were chosen that may have both literal (taking concrete objects, e.g., “to throw a stone”) and metaphorical or figurative use (taking abstract objects, e.g., “to throw an idea”). For each condition, half of the sentences were semantically plausible and the other half were anomalous (concrete object: “to throw the engine”; abstract object: “to throw the lesson”). The congruency of the combination depended on the compatibility of the verb's semantic constraints limiting possible arguments, and the semantic characteristics of the object.

Specifically, the figurative use of the verb was identified with the abstract-congruent condition. In fact, only in this condition was the verb used in its (conventional or easily conceivable) figurative meaning. Incongruent combinations were of such a type that (also according to the “career of metaphors” theory) would not be recognized as any known (conventional and lexicalized) metaphor or idiom and, in the absence of a context that could give hints for a possible “novel” metaphorical interpretation, could probably be processed as (wrong) instances of the literal meaning of the verb (e.g., compare “to throw an idea” with “to throw a lesson”). The experimental design, where nouns and objects were the same in the congruent and incongruent condition and only combinations changed, allowed not only comparison of the concrete and the abstract domain, but also control for variables that were described to affect behavioral and psychophysiological data on the processing of figurative language (AoA, familiarity, context, etc.; see [74, 75]).

What are the predictions deriving from the complex set of distinctions that have been found to characterize the processing of abstract and figurative language? One could observe that the probabilistic distribution of the verb object is likely to follow a decreasing pattern from literal (concrete object) to figurative (abstract object) to (possibly) implausible concrete objects and finally implausible abstract objects. The hypothesis thus could be that, in the general population, any component linked to low probability of the object following the verb (most probably, but not necessarily, an N400) should increase its amplitude from abstract to concrete and from plausible to implausible. Due to the dual nature of figurative verb-object combinations (which are at the same time more abstract, i.e., less likely to elicit concreteness-linked N400, and figurative, i.e., more likely to elicit figurative language-linked N400), the two effects could be in competition, but the scalp localization of N400 should help disentangle them.

As to the clinical groups in the present study, it could be predicted that the group of children with the most “crucial” impairment (sensory-motor skills according to embodiment theories or linguistic skills according to the language-based hypothesis) will show the greatest impairment with abstract words. SLI children, whose impairment especially affects functions located in LH areas, may be less sensitive to the plausibility and probability characteristics of the linguistic structures they are exposed to, and therefore they might show reduced ERP components after violations. Alternatively, it could be the case that SLI children have similar, or even larger, responses to violations in the thematic structure of the verb (or to its use in a figurative manner) and that their difficulties only relate to the cognitive process of explicit judgment of the linguistic structures, that is, to a metalinguistic deficit. NVLD children, on the other hand, may be less sensitive to concreteness effects and should show reduced responses to concrete words, since their weakest functions are those related to RH activity. The integrated analysis of behavioral data, ERP components, and their localization may give hints as to the processing of concrete and abstract stimuli in children with developmental impairments and also to the best models for figurative language processing and representation. Among others, the dual-coding theory predicts that SLI children have relatively greater difficulties with abstract words and NVLD children with concrete words. The context availability theory, by contrast, would not predict that particular differences emerge between the two clinical groups.

## 2. Method

### 2.1. Participants

A total of 40 native Italian speaking children, ranging in age between 6 and 15 years, participated in the study. The sample included 12 children with SLI, 13 children with NVLD, and 15 typically developing (control) children. The participants with SLI and NVLD were recruited after a thorough selection of medical records of patients diagnosed at the research hospital, based on specific criteria. The inclusion criteria for the participants with SLI were as follows:

- A diagnosis of language impairment.
- Absence of perceptual, visual-spatial, and/or praxic impairments.
- Full scale IQ greater than or equal to 85 (with a tolerance of 2 points) at the Wechsler Scales (WPPSI or WISC-R).
- Performance IQ higher than 90.
- A score significantly below average in at least two of the following linguistic tests (of which one is at least lower than two standard deviations and another one is at least lower than one standard deviation below age means): the British Picture Vocabulary Scale (BPVS [76]), the Token test for children [76], a test of grammatical comprehension for children (Test di Comprensione Grammaticale per Bambini, TCGB [77]), a naming task (Denominazione di Sostantivi [76]), and a sentence repetition task (Ripetizione di frasi [78, 79]). The Italian versions and norms of these tests can be found in Fabbro [80].

The children with NVLD were one-to-one matched with those with SLI, on the basis of gender, chronological age (+/− 6 months), and full scale IQ. The inclusion criteria for the NVLD group were as follows:

- A diagnosis of specific developmental impairment (e.g., Developmental Motor Coordination Disorder, Mixed Specific Developmental Disorder, and Specific Learning Disorder) with evident difficulties in nonverbal skills not associated with other neuropsychological disorders.
- Absence of linguistic problems.
- Full scale IQ greater than or equal to 85 (with a tolerance of 2 points) at Wechsler Scales (WPPSI or WISC-R).
- A discrepancy of at least 15 points between Verbal IQ and Performance IQ.
- A score significantly lower than average (scaled score ≤ 7) in at least two of the following subtests of the WISC-R: picture completion, block design, object assembly, and mazes.

For both clinical groups, the presence of autistic spectrum disorders had been assessed through questionnaires and structured interviews with parents and excluded according to standard DSM-IV criteria. The typically developing children (control group), without any documented deficits in linguistic and nonverbal abilities, were recruited in local schools and matched one-to-one with each pair of participants (SLI and NVLD) in the clinical population groups, on the basis of gender and chronological age (+/− 6 months). The IQ of typically developing children was estimated through the administration of five WISC-R subtests (picture completion, block design, object assembly, vocabulary, and digit span) in order to exclude previously unidentified cognitive deficits. All children underwent further testing on a test of morphosyntactic abilities (Clitic repetition, [81]).

The study was approved by the Ethical Committee of the Research Institute according to standards of the Helsinki Declaration (1964), and informed consent was signed by all children's parents.

### 2.2. Materials and Procedure

The performances of the two clinical groups (SLI and NVLD) and of control children were compared for both behavioral and ERP data. The task assessed the ability to judge the semantic plausibility of verb-object (infinitive verb) combinations. One hundred and twenty stimuli were created, belonging to four different groups of sentences: 30 congruent combinations of a verb with a concrete object (e.g., “to prepare a coffee”); 30 congruent combinations of the same verbs with an abstract object (e.g., “to prepare a plan”); 30 incongruent combinations of the verb with an implausible concrete object (e.g., “to prepare the rain”); 30 incongruent combinations of the verb with an implausible abstract object (e.g., “to prepare a science”). Use of the definite or indefinite article before the noun was balanced across sentences and their use was always acceptable according to language rules in Italian (so that no syntactic violations were present in the sentences). The unacceptable (incongruent) sentences were incongruent on grounds of their violating some of the semantic constraints of the verb (e.g., neither science nor rain can be prepared, as “prepare” implies that the object is something that can be manipulated and cannot be applied to external, uncontrollable events or phenomena). They included light verbs (bring, do, take, etc.), motion verbs (go, run, follow, overcome, etc.), and other verbs that are frequently used in both a literal and a figurative way. Some combinations were very frequent idiomatic expressions used in Italian (e.g., “take the sun,” i.e., “to sunbathe”). Each of the verbs was used once in each kind of combination, and each object was used once in a congruent and once in an incongruent combination. This means that the verb stimuli in the concrete and in the abstract condition were identical, excluding any difference due to contextual factors. In the congruent and incongruent conditions, moreover, also the nouns (the verb arguments) were identical, allowing perfect comparability of the sets of stimuli (except for the congruency of the combination). Given the experimental design, a comparison of the abstract-congruent versus abstract-incongruent condition allowed a perfect control of all lexical variables related to the verbs and to the nouns (which were all identical but combined in different ways). Any difference in ERP responses to congruent and incongruent combinations (not distinguishing between concrete and abstract objects) as well as any difference between concrete and abstract conditions (without distinguishing between congruent and incongruent objects) would thus reflect the sum of two effects, due to concreteness and to congruency. A “pure” effect of concreteness could emerge from the comparison of concrete- and abstract-incongruent combinations, whereas a “pure” effect of figurativity would emerge from the comparison of congruent and incongruent abstract objects (the congruent objects being the only ones which could allow a figurative reading of the verb). Therefore, the interaction of concreteness with congruency and its further modulations related to group differences will be the main concern of the present study.

The concreteness level of all nouns had been judged by ten adult Italian speakers, on a seven-point Likert scale ranging from “fully abstract” (1) to “fully concrete” (7). Words with a mean score lower than 4 were considered as abstract objects, whereas words with a mean score equal to/greater than 4 were considered as concrete objects. Each noun appeared as object twice, once in an acceptable sentence and once in an unacceptable sentence. Abstract and concrete words were balanced according to frequency and Age of Acquisition [82]. The selected sentences were read by a female voice and digitally recorded. The verbs and the objects were recorded separately and then combined (Praat software [83]), so as to ensure comparability in sentence duration and prosody. Congruency and imageability of the verb-object combinations were rated by 23 native Italian adult speakers (imageability was rated on a 7-point Likert scale, from “very difficult to imagine” to “very easy to imagine”; congruency was rated on a dichotomous scale as “congruent, you can say it” or “incongruent, you cannot say it”). Mean imageability ratings for abstract combinations were 3.3; for concrete combinations, 4.5 (*p* = .001). As to congruency, only verb-object combinations with at least 75% agreement about their congruency (i.e., their being or not being acceptable) were included in the final list of stimuli. For congruent combinations, the mean percentage of “acceptable” ratings was 96.96% (SD = 5.39), while for incongruent combinations it was 6.3% (SD = 7.22), *p* < .001. (Fourteen adult subjects were further asked to rate all the 60 incongruent (i.e., previously classified as such based on dichotomous judgments) combinations as (1) easy, (2) difficult, or (3) impossible to interpret and to write the interpretation when not impossible. Only 3.8% of the total set of ratings for the incongruent sentences suggested that they were “easy to interpret” (rating = 1). The remaining ratings (96.2%) indicated that the combinations were either “difficult to interpret” (40%) or “impossible to interpret” (56%). Analyzing abstract and concrete combinations separately, these percentages are impossible = 44% and 68%; difficult = 52% and 28%; easy = 4% and 3%, respectively. This confirms that combinations with abstract verbs lend themselves more easily to figurative interpretations but also that the combinations that were classified as incongruent were indeed judged as difficult or impossible to interpret in the great majority of cases.) The list of stimuli is reported in the Supplementary Materials available online at http://dx.doi.org/10.1155/2015/475271.

#### 2.2.1. Data Recording and Preprocessing

The stimuli were stored on a PC and presented using STIM2 software package (Neuroscan) via headphones (Sennheiser HD270), at a comfortable volume of 80 dB. During the experiment, participants listened to the sentences in a quiet room. They were instructed to listen carefully to the sentences, in order to judge their acceptability; the exact instruction, simplified to be understood by children, was “can you say xxx (verb infinitive) yyy (object)?” (e.g., “can you say ‘to throw an idea'”?). The congruency judgments were manually recorded by the experimenter, and the total number of correct judgments was computed for both congruent and incongruent sentences in each condition (concrete-congruent, concrete-incongruent, abstract-congruent, and abstract-incongruent).

EEG data were recorded from 19 standard scalp locations of the standard 10–20 system at a sampling rate of 1000 Hz and referenced to the left and right mastoid. All artifacts (eye movements, head movements) were excluded if the standard deviation of the channel exceeded 100 mV. ERP averages time-locked to the beginning of the critical word (the verb's object) were computed with a 100 ms prestimulus baseline and a 1000 ms ERP time window. Electrode impedance was kept below 10 kΩ. All electrodes were connected to a Neuroscan amplifier (SynAmps vers. 1). The electrophysiological signals were sampled at 1000 Hz rate and then filtered via a zero-phase bandpass procedure (0.3–40 Hz). The continuous EEG signal was treated with an automatic rejection criterion applied to all the electrodes (sections exceeding 70 *μ*V were excluded). The ERP recordings of 4 children had to be discarded because of the presence of too many artifacts.

### 2.3. Data Analysis

#### 2.3.1. Analysis of Behavioral Data

The total sample of behavioral data included 37 children (resulting from the exclusion of 3 participants), whose characteristics are described in Table 1. The performances on the experimental task were analyzed by means of a repeated measures ANOVA, considering group (i.e., SLI, NVLD, and control) as a between-subjects factor and both concreteness (i.e., concrete versus abstract) and congruency (congruent versus incongruent) as within-subjects factors. Significant differences were further analyzed by post hoc tests. Finally, Pearson's correlations were computed between some of the variables expressing relevant characteristics of the children's functioning (Verbal and Performance IQs, scores on language and visual-spatial tests) and both behavioral (accuracy) and ERP results. Special attention was given to the correlations involving abstract-congruent combinations, since these were considered as instances of the figurative use of verbs. Only age-standardized scores (IQ scores, *z*-scores, or Wechsler's weighted scores) were used, in order to avoid spurious correlations due to age effects.

#### 2.3.2. ERP Analysis 1: Region of Interest (ROI) Analysis of ERPs

The database included ERP recordings from 36 children (resulting from the exclusion of 4 participants). Their characteristics are described in Table 2. For ROI statistical analysis of ERPs, a repeated measures ANOVA was conducted with group (NVLD, SLI, and controls (CNTR)) as between-subjects factor and three within-subjects factors: concreteness (abstract or concrete), congruency (congruent or incongruent), and ROIs (left, central, or right). Electrodes were grouped into three separate Regions of Interest (ROIs): left (F7, F3, T7, and C3), central (FZ, CZ, and PZ), and right (F4, F8, C4, and T8), and differences between mean amplitudes among different conditions and between groups were tested. The time window for statistical analyses was 300 to 500 ms, namely, the N400 time window based on our experimental question.

#### 2.3.3. ERP Analysis 2: SPM-ERP Preprocessing and Statistical Analysis

A further complementary analysis was performed using the “SPM-ERP” method implemented in SPM (Statistical Parametric Mapping) (http://www.fil.ion.ucl.ac.uk/spm/software/spm8/), applying Random Field Theory (RFT) [84] to EEG data. For every subject continuous EEG file (*∗*.cnt) was first converted to a SPM file in order to perform preprocessing steps of all data. A high pass filter (0.5 Hz) and a low pass filter (30 Hz) were applied to the continuous EEG data file for each subject. Epochs of 800 ms after stimulus onset were computed with an additional 100 ms prestimulus baseline. All artifacts (eye movements, head movements) were excluded if the standard deviation of the channel exceeded 100 mV. After the artifacts rejection procedure, all files were additionally inspected in order to check whether some of the trials classified as “bad trials” could be reincluded in the analysis. Subjects with more than 50% of bad trials were excluded from the statistical analysis. Finally, only seven subjects for each group were included for purposes of the SPM-RFT statistical analysis (see Table 3 for subsample characteristics). The mean number of valid trials for all subjects was 91,89 (percentage of accepted trials was 76.6%) and no differences emerged between the three groups (*F*(2,19) = 0,806, *p* = .462).

Statistical analyses of ERPs at the group level were performed according to the SPM procedure [85]. First, ERP amplitudes for each subject and each condition were transformed into scalp maps of 64 × 64 pixels using a two-dimensional (2D) linear interpolation (i.e., a transformation based on electrode coordinates in the Montreal Neurological Institute (MNI) space). Second, scalp maps of ERP amplitudes were concatenated over time to produce a three-dimensional (3D) volume (2D space × time).

In order to investigate differences among conditions we used the simple main effects method [86] to obtain contrasts between levels of a variable within all other levels of the other variable in an interaction. Differences in mean amplitudes between different conditions within or between different groups were tested by means of Paired-Sample *t*-tests (differences between conditions within groups) and Two-Sample *t*-tests for independent samples (differences between groups within conditions). Therefore, comparisons between conditions or between groups were carried out as linear contrasts testing (1) differences within incongruent sentences between the abstract and concrete conditions (i.e., concreteness effect) and (2) differences within the abstract or concrete domain between incongruent and congruent sentences (i.e., congruency effects and figurative language effects).

The time window for statistical analysis was 300 to 500 ms. Given our a priori hypothesis and the very strict selection criteria for trials to be included (that lead to the inclusion of only 7 participants per group), results were assessed at a more liberal statistical threshold (*p* < .05) uncorrected for voxels (a voxel in this context is a data point defined by location and latency, e.g., electrode F4 at latency 400 ms), while clusters refer to electrodes adjacent to each other in space and time, as illustrated in SPMs.

The results will be analyzed in terms of the three effects that were the focus of the present study, namely, (a) a concreteness effect, which predicts that concrete targets elicit larger N400, especially over (RH according to some of the studies) central-anterior areas, possibly delayed due to the younger age of participants and possibly prolonged over the typical N400 window; (b) a figurative language effect, predicting that figurative expressions elicit larger N400; (c) a congruency effect, predicting that incongruent combinations should elicit larger N400 responses over central but also posterior, medial-temporal areas.

## 3. Results

### 3.1. Behavioral Data

**Figure 1:**
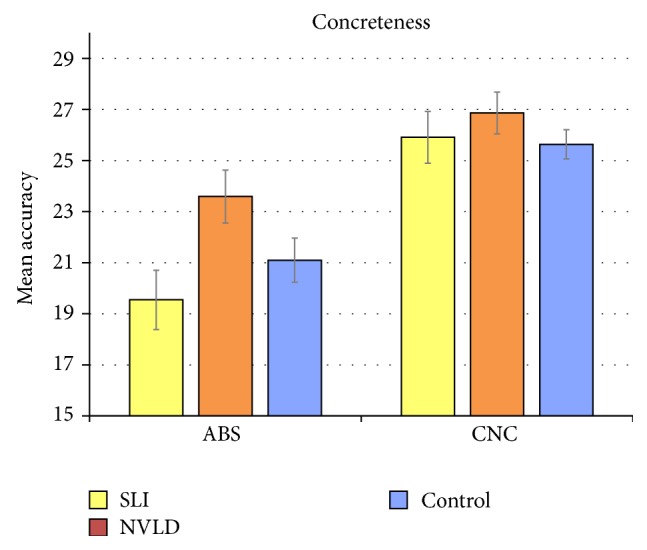
Mean accuracy scores for concrete and abstract verb-object combinations in the three groups. Error bars represent standard errors.

**Figure 2:**
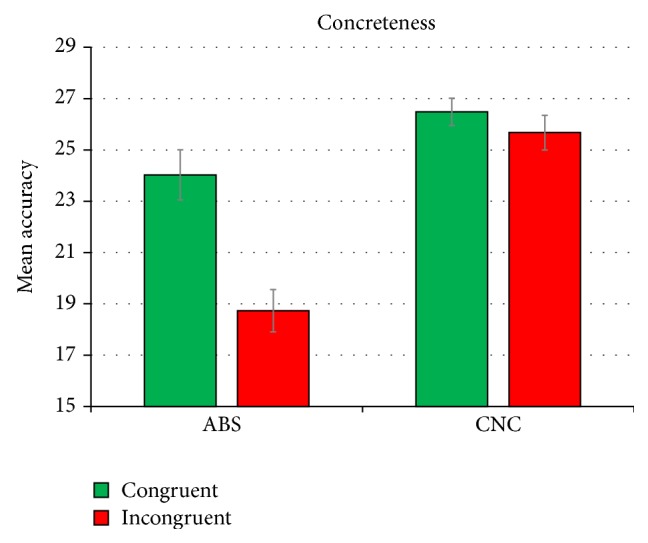
Mean accuracy scores for the concreteness × congruency interaction in the whole group. Error bars represent standard errors.

The 120 responses obtained from each participant were analyzed in terms of accuracy. As reported in the previous section, the total sample for behavioral test analyses is of 37 participants (see Table 1). From a repeated measures ANOVA with clinical group as between-subjects factor (3 groups × 2 concreteness levels × 2 congruency levels), significant effects emerge for concreteness, *F*(1,34) = 93.457, *p* < .001, *η*_*p*_^2^ = .733, and congruency, *F*(1,34) = 10.647, *p* = .003, *η*_*p*_^2^ = .238. All participants, thus, had more difficulties responding to abstract sentences and to sentences (both concrete and abstract) of the congruent type. The interaction group × concreteness (see Figure 1) is close to significance, *F*(2,34) = 3.081, *p* = .059, *η*_*p*_^2^ = .153, while the interaction group × congruency is not. As shown in Figure 2, SLI participants performed significantly lower (mean = 19.545; sd = 2.859) than NVLD (mean = 23.591; sd = 3.441) participants in processing abstract sentences only (*p* = .008), *η*_*p*_^2^ = .331.

A further significant concreteness × congruency interaction (see Figure 2) emerges, *F*(1, 34) = 20.914, *p* < .001, *η*_*p*_^2^ = .381, highlighting a general disadvantage in response to abstract sentences of the congruent type. In fact, responses to abstract objects of the congruent type are significantly less accurate (mean = 18.73; sd = 5.947) than responses to abstract objects of the incongruent type (mean = 24.03; sd = 5.019) (*p* = .002).

The analysis of correlations shows a positive relationship between accuracy scores for abstract-incongruent sentences and Verbal IQ, *r*(37) = .491, *p* = .002. Verbal IQ also correlates with concrete-congruent combinations (*r* = .376, *p* = .022) and with all accuracy scores in averaged conditions: all abstract, all congruent, all concrete, and all incongruent combinations as a whole (*r*s between .333 and .518, *p*s < .044). Performance IQ, by contrast, fails to show any significant correlation. Further correlations in the whole group emerge, involving only expressions of the abstract, incongruent type. These correlations include Clitic repetition (*r* = .494, *p* = .002), syntactic comprehension (Token test, *r* = .408, *p* = .032), and vocabulary and digit span subtests from the WISC-R (*r* = .333, *p* = .044 and *r* = .359, *p* = .029, resp.); moreover, there is a negative correlation of abstract combinations with the mazes subtest (*r* = −.369, *p* = .025). Scores at the vocabulary subtest also correlate with abstract (*r* = .427, *p* = .008) and with congruent combinations (*r* = .343, *p* = .038) as a whole. Finally, Clitic repetition correlates with incongruent combinations as a whole (*r* = .396, *p* = .015). No significant correlations emerged for any subtest of the Performance Scale. A further analysis was performed, computing correlations within each subgroup. Considering only the correlations that are specific (exclusive) for abstract (congruent or incongruent) sentences, this analysis showed that accuracy in control children is associated with pattern copy (VMI, with abstract-incongruent sentences, *r* = .586, *p* = .022) and visual-constructive skills (block design subtest, with abstract-incongruent sentences, *r* = .540, *p* = .038), but also with Clitic repetition (with abstract-congruent combinations, *r* = .609, *p* = .016). NVLD children's accuracy is associated with Performance IQ (*r* = .526 with abstract-congruent and .784 with abstract-incongruent combinations, resp.). SLI children showed only a quite specific correlation with Verbal IQ (with abstract combinations, *r* = .752, *p* = .008), although all linguistic tests tended to positively correlate with several accuracy scores (both abstract and concrete), especially for incongruent combinations.

#### 3.1.1. Region of Interest (ROI) Analysis of ERPs

**Figure 3:**
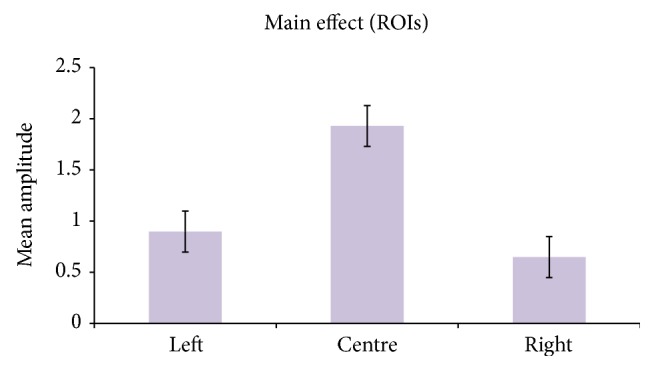
Mean amplitudes of ERPs elicited in the left, central, and right ROIs, in the whole group. Error bars represent standard errors.

Analysis of the ERPs, time-locked to the abstract or concrete target words (which were either congruent or incongruent with respect to the preceding verb), showed a significant main effect of ROI (*F*(2,66) = 27,160; *p* < .001), two two-way interactions between group and concreteness (marginally significant, *F*(2,33) = 3, 185; *p* = .054) and between group and congruency (*F*(2,33) = 4, 822; *p* = .015), and a particularly interesting four-way interaction between group, concreteness, congruency, and ROI (*F*(4,66) = 2, 763; *p* = .035). Post hoc analysis on the mean amplitudes for each of the three ROIs revealed that the positivity elicited at the central electrodes was significantly larger than in both the left (*p* < .001) and right (*p* < .001) ROIs (see Figure 3).

**Figure 4:**
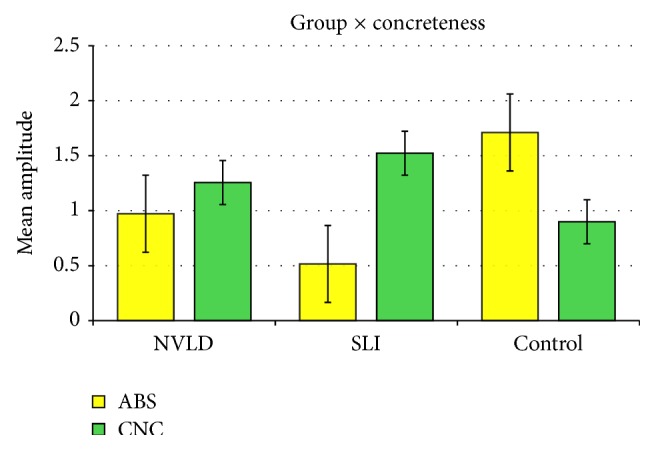
Mean amplitudes of ERPs elicited by concrete and abstract stimuli in the three groups. Error bars represent standard errors.

In order to investigate the nature of these interaction effects, we used the method of simple main effects [86] to obtain contrasts between levels of a variable within all other levels of the other variable in an interaction. Pairwise multiple comparisons carried out in order to break down the interaction group × concreteness revealed a reduced positivity (mean difference = −1,196; *p* = .039) for abstract target words for the SLI group versus control (CNTR) while no significant difference emerged when comparing NVLD to controls or NVLD and SLI participants on abstract verb objects. On the other hand, for concrete words, no significant difference emerged between the three groups (see Figure 4).

**Figure 5:**
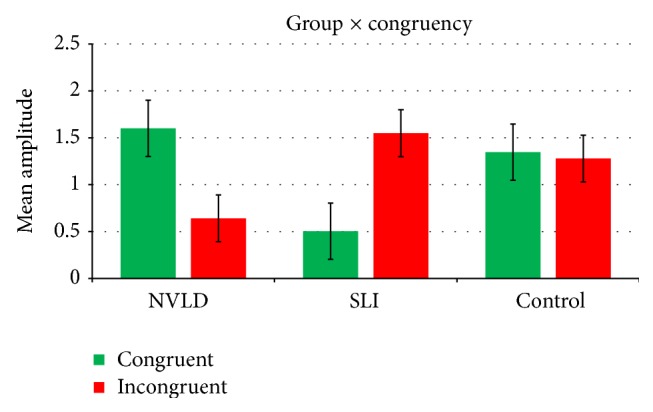
Mean amplitudes of ERPs elicited by congruent and incongruent stimuli in the three groups. Error bars represent standard errors.

Pairwise *t*-tests performed to decompose the second two-way interaction group × congruency (see Figure 5) highlighted instead a significantly increased positivity (mean difference = 1,094; *p* = .043) in the NVLD group when compared with SLI children for objects congruent with the preceding verb. No difference emerged when comparing each group to the control subjects. For incongruent targets, no significant differences emerged between the three groups.

In order to better clarify differences between the three groups of children, the four-way interaction was broken down and pairwise multiple comparisons were carried out. For abstract-congruent objects (representing figurative use of verbs) results revealed a significant difference in terms of reduced positivity distributed over the left (*p* = .037) and midline portions (*p* = .046) of the scalp for children with SLI compared to controls. By contrast, a significant difference was found in response to abstract-incongruent objects for both NVLD (*p* = .042) and SLI (*p* = .038) when compared to controls, in terms of reduced positivity distributed over the left electrodes only. On the other hand, differences between the three groups were found for concrete objects, in terms of increased positivity. For concrete-congruent objects, an increased positivity distributed over the midline electrodes was found for the NVLD group (*p* = .037) with respect to controls. Furthermore, a nearly significant difference was found in response to concrete-incongruent objects between the SLI and the NVLD group, in terms of increased positivity distributed over the left (*p* = .076) and the midline portions (*p* = .061) of the scalp.

### 3.2. SPM-ERPs

#### 3.2.1. Concreteness Effect: Abstract-Incongruent versus Concrete-Incongruent

**Figure 6:**
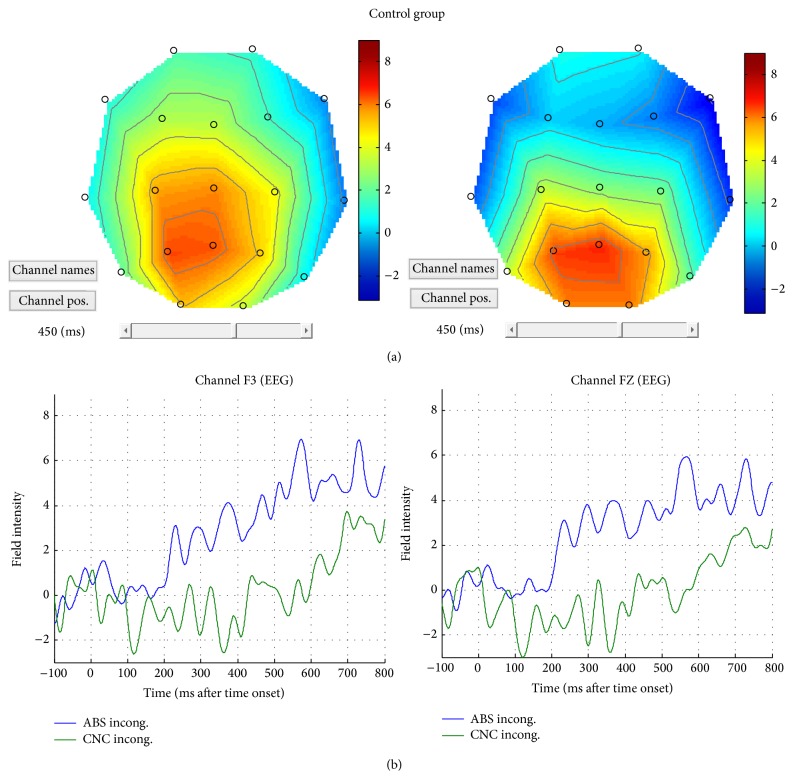
(a) Pattern of brain activity for abstract-incongruent and concrete-incongruent combinations. (b) ERP responses to abstract-incongruent (blue line) and concrete-incongruent (green line) objects for the control group, on left and central sites.

**Figure 7:**
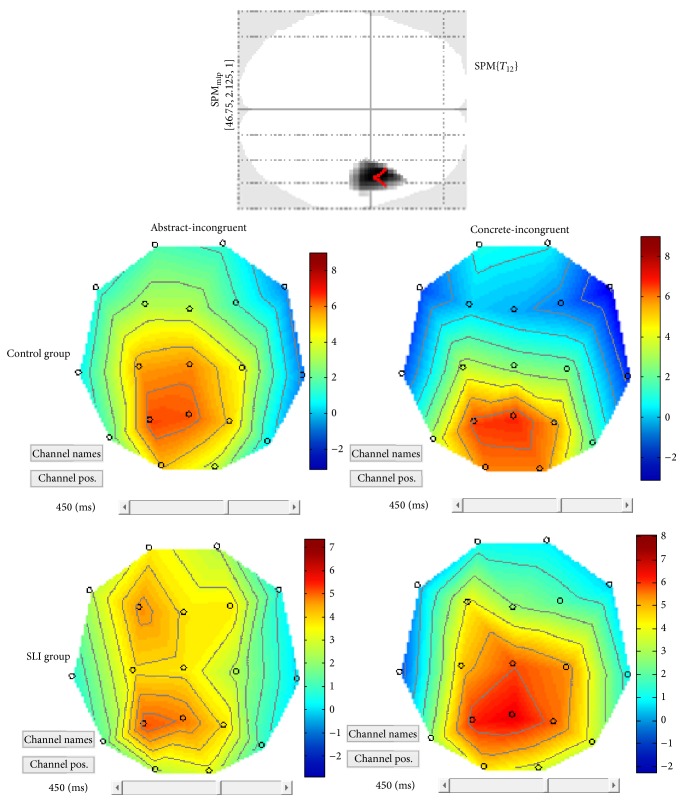
Patterns of brain activation in response to abstract- versus concrete-incongruent stimuli, in the control group and in the SLI group.

This contrast revealed a difference in right anterior areas for the control group only. Concrete-incongruent targets elicited a larger negativity in anterior areas bilaterally (i.e., frontal areas) with respect to abstract-incongruent targets (see Figure 6). When assessing the “concreteness effect” contrast between groups, a difference emerged only between control and SLI children (Figure 7). Particularly, when control children were compared to children with SLI (CNTR > SLI), an effect over right frontotemporal electrodes was found. This is due to the larger negativity in control children for concrete-incongruent targets in comparison to abstract-incongruent targets. This effect is more lateralized over right anterior electrodes. Notably, no main effect of concreteness was found within the SLI group, suggesting that a concreteness effect was present only in control children.

#### 3.2.2. Congruency Effect within Concrete and Abstract Targets

**Figure 8:**
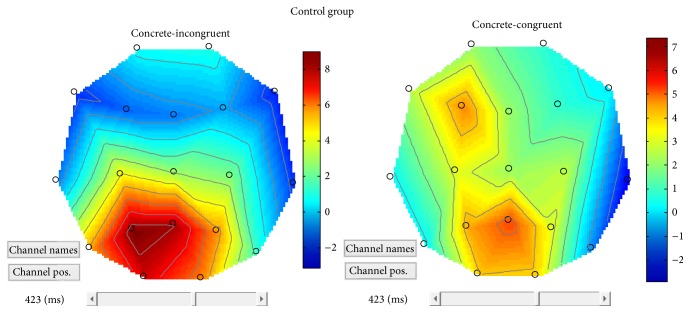
Patterns of brain activation in response to concrete-congruent versus concrete-incongruent stimuli, in the control group.

Considering congruency effects in the concrete dimension (i.e., concrete-incongruent versus concrete-congruent) within each group, a main effect of congruency emerged within the control group in terms of larger positivity in peak amplitudes for incongruent objects compared with congruent objects over left posterior electrodes. From the Field Intensity Maps (see Figure 8), moreover, a larger anterior negativity appears bilaterally for incongruent objects when compared with congruent objects.

**Figure 9:**
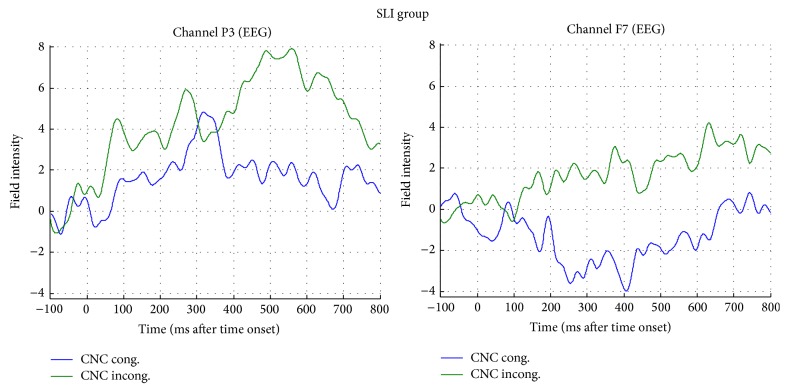
ERP recordings corresponding to concrete-congruent and concrete-incongruent stimuli in the SLI group, in left parietal (P3) and frontal (F7) sites.

A main effect of congruency in the concrete condition for the SLI group appeared over the posterior-central electrodes bilaterally and over the left anterior electrodes. For concrete-incongruent objects compared to concrete-congruent objects, an increased positivity was found over central-posterior area and in left frontal areas (see Figure 9), a reversed congruency effect.

**Figure 10:**
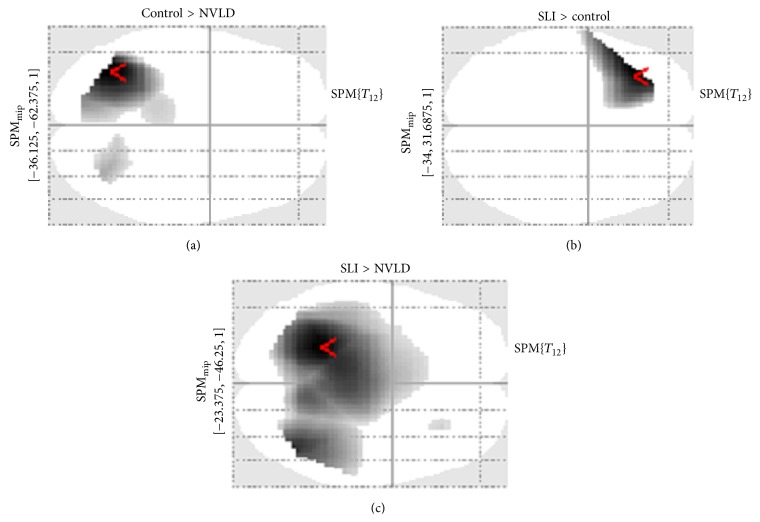
Across-groups SPM contrasts. (a) Control group versus NVLD. (b) SLI versus control. (c) SLI versus NVLD.

Possible differences in the congruency effect within the concrete domain (concrete-incongruent versus concrete-congruent) were then explored between the three groups. The results pointed out a difference over the posterior-central electrodes between the control group and the NVLD group (CNTR > NVLD), since the congruency effect in NVLD led to a reduced positivity in posterior-central regions with respect to controls (see SPM Maximum Intensity Projection (MIP) in Figure 10(a)). On the other hand, when SLI children were compared to controls (SLI > CNTR), a larger positivity (or reduced negativity for SLI group) emerged over the left frontal electrodes in response to concrete-incongruent versus concrete-congruent objects (see SPM MIP in Figure 10(b)). Finally, comparing SLI with NVLD, a difference was found over the central-posterior electrodes bilaterally, indicating a larger (but reversed) congruency effect within the concrete domain for SLI children (see SPM MIP in Figure 10(c)).

**Figure 11:**
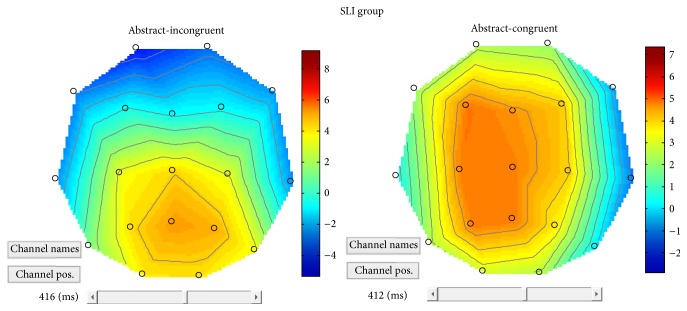
Patterns of brain activation in response to abstract-incongruent versus abstract-congruent stimuli, in the SLI group.

**Figure 12:**
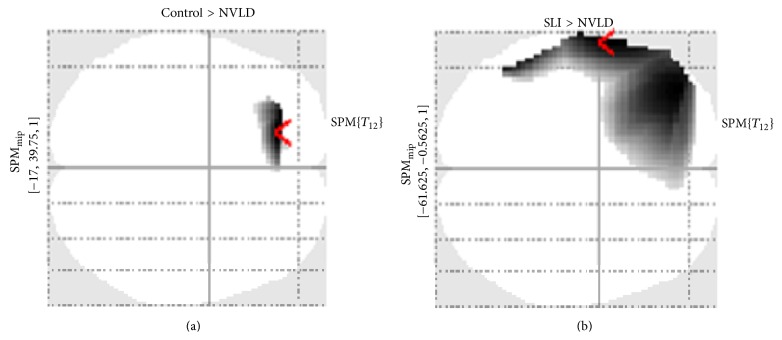
SPM contrasts for the congruency effect within abstract trials, in the two clinical groups.

Within the abstract domain, a congruency difference (expressing also a figurativity effect) was found only within the SLI group, in terms of larger positivity for abstract-incongruent targets compared to abstract-congruent targets (see Figure 11). For the CNTR and the NVLD group, no main effect of congruency was found within the abstract dimension. When comparing the control group with the NVLD group, a difference was found over left frontal electrodes (see SPM MIP in Figure 12). Comparing the two groups with SLI and NVLD, a difference emerges over left temporofrontal electrodes (see SPM MIP in Figure 12).

**Figure 13:**
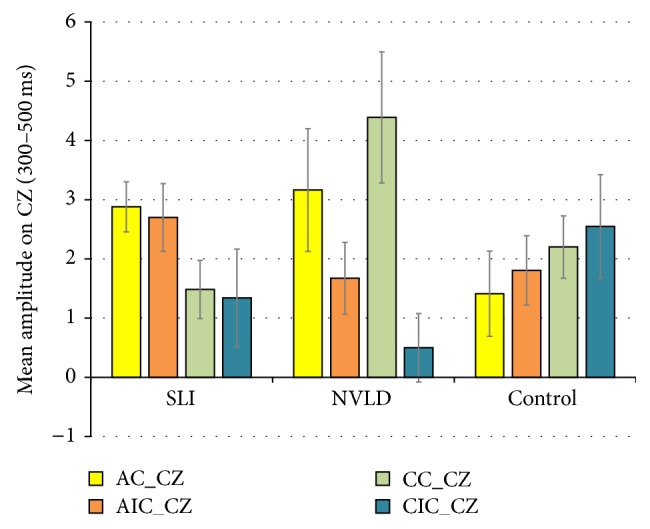
Mean amplitudes of ERP responses in the four conditions, for the three groups, on central sites (300–500 ms period), summarizing concreteness (AIC versus CIC), congruency (CC versus CIC), and figurativity (AC versus AIC) effects. AC: abstract-congruent; AIC: abstract-incongruent; CC: concrete-congruent; CIC: concrete-incongruent. Error bars represent standard errors.

ERP responses at CZ to the four types of verb-object combinations are represented in Figure 13, in order to summarize the three effects (concreteness, congruency, and figurativity) in the three groups.

#### 3.2.3. Correlations between N400 Amplitude and Behavioral Tasks

Correlations were computed for ERP amplitudes at CZ (in the temporal window between 300 and 500 ms) with accuracy scores, as well as with performance on cognitive and linguistic tasks (age-standardized scores). No significant correlations emerged between behavioral and ERP data from the experimental task in the whole group. However, interesting correlations emerged with ERP amplitude in the three groups separately: in the SLI group only, ERP responses are associated with behavioral data (accuracy). Specifically, ERPs correlate with behavioral responses for abstract combinations (*r*(12) = .638, *p* = .035) and for concrete-congruent combinations (*r* = .718, *p* = .013). Further interesting correlations include, in the whole group, a positive association between ERP responses to abstract-congruent combinations and VIQ (*r*(36) = .353, *p* = .035) and a negative correlation between ERP responses to concrete-congruent combinations and PIQ (*r* = −.397, *p* = .016); in the control group, a positive association of ERP responses with abstract-congruent (figurative) combinations and full scale IQ (*r*(13) = .643, *p* = .018); in the NVLD group, a close-to-significant negative association with PIQ (*r*(11) = −.536, *p* = .089); in the SLI group, a positive association with VIQ (*r*(12) = .577, *p* = .05).

## 4. Discussion

### 4.1. Discussion of Behavioral Results

#### 4.1.1. Differences among Combination Types (Abstract, Concrete, Congruent, and Incongruent)

With respect to behavioral results, a general disadvantage emerged for all children in response to abstract combinations of the congruent, acceptable type. Since the difference between responses to concrete-congruent and concrete-incongruent sentences is not significant, it can be concluded that the specific difficulty for responding to acceptable sentences (i.e., to recognize them as acceptable) is due to figurative sentences only (i.e., it is the figurative use of verbs that makes it difficult to recognize the sentence as an acceptable one). This result is in line with data in the literature, showing that judging figurative sentences is more difficult than judging literal/concrete expressions (e.g., [19, 87]) and that judging congruent, acceptable sentences is more difficult than judging incongruent ones. Also Sabisch et al. [72] with both typically developing and language-impaired children found a higher proportion of correct responses with semantic violations than with correct sentences and argued that sentences containing semantic violations may be particularly salient because they are very uncommon; alternatively, these authors propose that there may be a specific difficulty to decide whether the expression is “uncommon” due to the figurative use of the verb (which would require a judgment of acceptability) or to an additional anomaly in the specific abstract object (which would require a judgment of unacceptability).

#### 4.1.2. Differences between SLI and NVLD Participants

A further important result is that SLI participants are generally impaired with respect to NVLD participants, more evidently so for the sentences including abstract object arguments. Therefore, the hypothesis of a reduced sensitivity to the characteristics of literal versus figurative use of verbs in language-impaired subjects finds support from the present data. Also Sabisch et al. [72] found that SLI children performed generally worse than control children and that the advantage for semantically incongruent sentences was more evident in the SLI group.

#### 4.1.3. Correlations

Precious information comes from the analysis of correlations for this task, indicating important relationships between indexes of linguistic competence and performance with abstract/figurative stimuli, as well as concrete expressions. Very interesting is the association of accuracy scores for abstract stimuli with both verbal and visual-motor skills in the control group. The positive associations emerging in the NVLD group with Performance IQ and in the SLI group with Verbal IQ suggest that the specific impairment in each group has an impact on their ability to process abstract and figurative expressions.

### 4.2. Discussion of ERP Results

In general, increases and deflections in posterior ERPs in the 300 to 500 ms window for the three groups are rather long-lasting and can more easily be described as modulations of brain electric activities rather than single, punctual components. This seems to be in line with Welcome et al.'s [88] study on adult subjects, showing that concreteness effects emerged later, with abstract items eliciting greater positivity for much of the 1500 ms period until response at posterior lateral electrode sites, and suggested that processing in the imagery system may be characterized by a different time scale than for the verbal system.

Overall, the analysis of ERP responses in the whole group confirms the validity of the experimental paradigm: the significant ROI main effect indicates that variation in positivity is especially detectable at central sites, suggesting that the component may be assimilated to an N400 effect. As is evident from Figure 13, the prediction of a gradual increase in ERP responses from abstract to concrete and from congruent to incongruent is perfectly fulfilled in the control group, while deviant patterns are found in the two clinical groups. Precisely, ERP recordings during the sentence plausibility judgment task confirm a general impairment of SLI subjects, who appear to be less sensitive to both concreteness and congruency effects. At closer inspection, though, ERP data show reduced components in SLI subjects with abstract objects and for congruent versus incongruent combinations. NVLD subjects, by contrast, show ERP anomalies related to concrete objects, that is, those inducing a literal reading of verbs. Notably, abstract-congruent (figurative) combinations were the ones for which significant differences were found in accuracy (behavioral data) as well.

The results can be analyzed in terms of the three effects that were the focus of the present study, namely, (a) concreteness effect, (b) figurative language effect, and (c) congruency effect.

#### 4.2.1. Concreteness Effects

As to the concreteness effect, it can be observed that children with SLI are the least sensitive to this kind of manipulation. Indeed, this appears to depend on abstract objects evoking less positive responses, so that the difference between concrete and abstract targets turns out to be less negative. It could be concluded that, for SLI children, it is abstract words, and not concrete words, that evoke N400-like responses, suggesting a difficulty in the processing of abstract words. This effect is especially evident in LH areas, which suggests that this area is crucial for the processing of abstract words and expressions. If only incongruent combinations (for “purer” concreteness effect) are taken into consideration, only control children showed concreteness effects in terms of increased negativity over RH regions, and this activation is significantly higher as compared to SLI children over RH frontotemporal sites. Indeed, the activation observed after concrete-incongruent expressions can be viewed as the sum of concreteness effects and congruency effects: none of them appears to be evident for children with SLI, while children with NVLD show more marked effects over LH and midline areas.

#### 4.2.2. Figurative Language Effects

Particularly relevant for this discussion is the comparison between abstract objects in congruent and incongruent combinations. In fact, only abstract objects in congruent combinations lend themselves to figurative interpretations, whereas abstract objects in incongruent combinations very likely appear simply as “awkward,” not evoking any metaphorical or figurative meaning of the verb. This contrast reveals significant differences not on posterior areas, as would have been predicted by a usual incongruence effect, but rather on left frontotemporal areas. Specifically, with abstract targets, differential effects are observed especially over LH areas and are significant for the control group but reduced for both the SLI and the NVLD group. This suggests that this area is specifically involved in the processing of figurative language.

Kutas and Federmeier [89] already suggested that the N400 represents quantitative rather than qualitative differences between literal and figurative language, with metaphors requiring more complex mapping and conceptual integration processes than literal language. The N400-like response in this context is sometimes regarded as a separable component—the “FN400” (frontal N400)—with a somewhat different scalp topography and an apparent link with familiarity parameters. Therefore, many studies associate FN400s with familiarity and LPCs with recollection (see [90]). However, in spite of objective topographical differences, no study has clearly dissociated the N400 from the FN400, which appear to be functionally similar [89]. The present results give indications as to the functional distinctiveness of such component.

#### 4.2.3. Congruency Effects

The relevant comparison for this effect is between concrete-congruent and concrete-incongruent sentences, which have a literal meaning and are not confounded by figurative language effects, nor by concreteness effects. First of all, incongruent expressions (with concrete objects) in control children seem to be associated with higher positivity in posterior LH areas but also increased negativity on anterior sites bilaterally, which can be described as an N400 component. Different from this pattern, a “reversed” congruency effect within the SLI group appeared with concrete objects: for incongruent targets an increased positivity was found over central-posterior area and increased positivity (instead of the N400 observed in control children) was found in left frontal areas. Moreover, the congruency effect within the concrete domain was greater for control children than for children with NVLD over the central-posterior electrodes bilaterally.

These results suggest that children with SLI have anomalous responses to incongruence, even when it concerns concrete expressions, which should be less difficult for them. Sabisch et al. [72] also found no N400 effect in SLI children in response to incongruence, due to a relatively large negativity for correct sentences, and suggested weaker lexical-semantic representations of the verbs and their selectional restrictions in children with SLI. They additionally found that, in both typically developing and language-impaired children, semantic violations elicited a broadly distributed, late positive ERP component. As the late positivity is assumed to reflect processes of sentential judgment [91], these differences were interpreted as evidence that more effort is required for language-impaired children to perform the plausibility judgment task; such interpretation could apply also to the present findings.

In typically developing children, no asymmetry was found over anterior sites, but an asymmetry emerged for positivity over posterior sites (more evident on the LH). Also Juottonen et al. [52] described the N400 component in response to incongruent stimuli in children as having the maximum amplitude at parietal sites and reported longer latency and greater amplitude for children than for adults; however, the congruency effect (N400) in their study was more evident over the right than left hemisphere, extending from the frontal to the parietal regions [52].

## 5. General Discussion

### 5.1. Abstract Language Processing: Lateralization and Involved Functions

The significant differences found for concreteness effects in the three groups, with SLI children showing the least accuracy and the most deviant ERP patterns in the processing of abstract and figurative expressions, lend support to theories assuming that linguistic processes are crucial to the representation and comprehension of abstract language. By contrast, children with nonverbal impairments, whose linguistic skills are comparable to those of the control group, even show (nonsignificantly) better performance at behavioral tasks than the control group itself.

This clearly speaks against a primary involvement of RH processes in the comprehension of abstract expressions. Altogether, the analysis of the lateralization of ERP effects in the three groups rather suggests that abstract expressions (including figurative language) are processed in the LH but that this kind of processing is particularly difficult for SLI children possibly because of their specific impairments affecting verbal functions.

On the other hand, concrete expressions tend to elicit more right-lateralized responses in control children. Crucially, these responses to concrete final objects are reduced in children with NVLD: for concrete-congruent expressions, which should elicit concreteness but not congruency effects, NVLD children show increased positivity, that is, a reduced N400.

The general correlation between accuracy scores for abstract, incongruent sentences and Verbal IQ (with higher VIQs for NVLD children with respect to SLI children) further confirms that linguistic abilities play a crucial role in abstract language comprehension, apparently more than nonverbal ones. This finding is similar to what was found by Sabisch et al. [72]: the correlation analyses, conducted with data from children in both groups, revealed that smaller N400 effects were associated with poorer verbal short-term memory capacity and poorer use of word knowledge in general.

### 5.2. Incongruence Processing: Lateralization and Involved Functions

The finding of a greater difficulty in correctly judging abstract-congruent rather than abstract-incongruent combinations also points to a specific difficulty in the processing of figurative language. This is particularly evident in children with SLI; however, the impairment of SLI children in detecting incongruence is evident not only with abstract, but also with concrete objects, excluding an interpretation of the results based only on the abstractness-concreteness distinction and/or on figurative language processing.

Notably, the present findings show that LH functions (linguistic processes) are crucial not only to the comprehension of abstract expressions, but also to the processing of incongruence, that is, to semantic analysis going well beyond the lexical level and affecting deeper levels of the processing of meaning.

### 5.3. Figurative Language Processing: Lateralization and Involved Functions

Finally, our results have clear implications also for the issue of the role played by RH in processing figurative and metaphorical expressions. In fact, deviant patterns of activation in the LH are associated with difficulties in understanding figurative or abstract expressions. Particularly interesting, in this perspective, is the finding of a frontal N400-like component associated with figurative language expressions (see [89]). These results seem to speak against the idea that figurative, nonliteral expressions are understood with the mediation of the RH through conceptual metaphors and that visual-spatial simulations are at the basis of the representation of such metaphors [6]. Rather, they are consistent with accounts pointing to the central role of the LH in figurative language processing (see also [92]). Nonetheless, NVLD participants' anomalous responses to incongruent expressions (both concrete and abstract) in terms of larger N400 amplitudes as compared to those observed in control children may suggest that impairments of visual-spatial functions (RH-based) do affect the processing of semantic aspects, probably requiring greater effort in the integration process, although this analysis results in positive outcomes at the behavioral level: accuracy scores for children with NVLD are definitely not inferior to those of typically developing children. In this perspective, the LH without a strong support of the RH (as probably happens in NVLD) may succeed in effectively processing the linguistic stimuli and their semantic characteristics, as shown by accuracy data, but at greater costs reflected in ERPs only.

Our results from correlation analyses show that wide-ranging linguistic skills such as syntactic comprehension and production, lexical skills, and verbal memory are predictive of the capacity to understand abstract and figurative language. This means that purely lexical accounts of figurative language comprehension are probably reductive. Also Henderson et al. [93] showed that sensitivity to semantic context relates specifically to listening comprehension, whereas absolute amplitude of negative ERP deflections relates specifically to decoding. Previous studies have shown that the N400 effect is sensitive to individual differences in comprehension skill in infants [94], school-age children [93], and adults [95]. Associations concerning N400 amplitude, on the other hand, point to Verbal IQ as the main correlate for sensitivity to semantic violations in the whole group, while mutually opposite correlations are found in the two clinical groups with nonverbal skills, showing that SLI children's (but not NVLD's) processing of figurative language follows a totally anomalous pattern, depending on the severity of their verbal impairment.

### 5.4. Theoretical Implications

According to the dual-coding theory, a language-based semantic system located in the left hemisphere would subserve both concrete and abstract concepts, and a nonverbal semantic system located in the right hemisphere would be especially devoted to the representation of concrete concepts. Consistently with this theory, Binder et al. [96] found that some of the RH regions were strongly activated by concrete words but not at all by abstract words, and other regions were activated equally by both word types. The present results also seem to favor this interpretation, since specific language impairments disrupting LH functionality interfere with abstract language processing more than RH-based impairments. Based on context availability theory [71], by contrast, concreteness effects are explained by positing a single mechanism for concrete and abstract words but more extensively activated by concrete words due to the richer “context” associated with such words. If this were true, both RH and LH dysfunctions should be expected to have an impact on abstract language processing. This prediction is not confirmed by the present results, where SLI children showed by far the greatest impairment with abstract expressions.

It is to be acknowledged that the many factors and variables involved in the present experiment offer a complex picture and are not always easy to disentangle. First of all, the limited number of participants in the study calls for caution in drawing general conclusions. While it was expected that the expression of concreteness and figurativity in ERP components could have been clearly distinguished based on scalp localization (with concreteness N400 more anteriorly localized than classical N400), the finding of left anterior effects for figurativity (also described by Coulson and van Petten [97], and consistent with Papagno et al. [98]), albeit interesting for its theoretical implications, has made distinction of the various effects even more difficult. Moreover, ERP differences due to age and clinical characteristics of the children may have introduced further variability and unpredictability in the present results. Nonetheless, the clear dissociations between conditions and groups, the replication of findings that had been described in previous studies with different populations, and the consistency of the whole pattern of results are encouraging with respect to the meaningfulness of the results and of their interpretation.

## 6. Conclusions

Altogether, the results of the present study highlight the crucial role played by linguistic processes (extending beyond simple lexical knowledge and encompassing semantic and syntactic skills) and by the left hemisphere in the representation and comprehension of abstract and figurative language. Right-hemisphere functions, by contrast, seem to be crucial for the processing of concrete language, as claimed by dual-coding models [70].

It is suggested that the LH, even without support by the RH (as is the case in nonverbal disabilities), may succeed in processing the linguistic stimuli and their semantic characteristics with unaltered accuracy, although at greater costs as reflected in ERPs. These results do not favor the idea that figurative, nonliteral expressions are mainly understood with the mediation of the RH through conceptual metaphors [6], although visual-spatial skills are indeed involved in the processing of such expressions.

The suggestions emerging from the present findings should be considered not only for their theoretical implications, but also for their clinical and practical value. Indeed, the clear indications of the negative impact of verbal impairments on the acquisition and use of abstract knowledge and in the comprehension of figurative language highlight the importance of early interventions supporting and compensating for the lack of spontaneous insight into fine-grained semantic content. Although difficulties with pragmatic and social communication are described for individuals with NVLD, such difficulties seem to lie at a more superficial level (possibly behavior based) than those observed in SLI children, which thus appear to be additional risk factors for negative social, emotional, and intellectual development and deserve full attention among rehabilitation goals.

## Supplementary Material

The table contains a list of all verbs used in the verb-object combinations, and (in the corresponding columns to the right) of the four objects that were combined with each of the verbs, subdivided into concrete and abstract. The upper noun in each cell represents the congruent object, whereas the lower noun represents the incongruent object, for each of the two subgroups. Verbs were used in their infinitive form, and objects were introduced by a determiner (either a definite or indefinite article), chosen so as to preserve grammatical plausibility and accuracy with respect to Italian rules and use.

## Figures and Tables

**Table 1:** Characteristics of participants included in the behavioral experiment. Standard deviations in parentheses.

	SLI	NVLD	*t*-test	Control	*t*-test
*N*	11	11		15	
Male	9	10		12	

Chronological age (yrs)	*M* = 9.74	*M* = 11.12	*p* = .456	*M* = 10.24	All *p*s > .682
	(2.3)	(2.9)		(2.74)	

Full scale IQ	*M* = 94.09	*M* = 94.0	*p* > .999	*M* = 112.8	SLI versus control; *p* = .005
	(8.83)	(8.93)		(18.68)	NVLD versus control; *p* = .004
Verbal IQ	*M* = 84.82	*M* = 106.64	*p* = .005	*M* = 108.27	SLI versus control; *p* < .001
	(13.03)	(10.32)		(19.02)	NVLD versus control; *p* = .960
Performance IQ	*M* = 105.91	*M* = 82.18	*p* = .002	*M* = 114.8	SLI versus control; *p* = .292
	(9.93)	(7.6)		(20.29)	NVLD versus control; *p* < .001

Clitic repetition (*z*-score)	*M* = .034	*M* = .967	*p* = .004	*M* = 1.06	SLI versus control; *p* = .001
	(1.1)	(.31)		(.16)	NVLD versus control; *p* = .921

Vocabulary subtest	*M* = 6.64	*M* = 11.64	*p* = .001	*M* = 10.0	SLI versus control; *p* = .015
	(2.38)	(2.91)		(3.16)	NVLD versus control; *p* = .335

Block design subtest	*M* = 10.36	*M* = 6.27	*p* = .004	*M* = 11.8	SLI versus control; *p* = .407
	(1.69)	(1.9)		(3.78)	NVLD versus control; *p* < .001

Object assembly subtest	*M* = 9.82	*M* = 5.91	*p* = .006	*M* = 10.73	SLI versus control; *p* = .682
	(3.34)	(2.17)		(2.65)	NVLD versus control; *p* < .001

**Table 2:** Characteristics of participants included in the ERP experiment. Standard deviations in parentheses.

	SLI	NVLD	*t*-test	Control	*t*-test
*N*	12	11		13	
Male	10	9		11	

Chronological age (yrs)	*M* = 10.23	*M* = 10.61	*p* = .948	*M* = 10.43	All *p*s > .984
	(2.75)	(3.12)		(2.9)	

Full scale IQ	*M* = 95.08	*M* = 95.73	*p* = .992	*M* = 115.5	SLI versus control; *p* = .001
	(9.09)	(7.39)		(18.29)	NVLD versus control; *p* = .002
Verbal IQ	*M* = 85.83	*M* = 107.91	*p* = .002	*M* = 111.23	SLI versus control; *p* < .001
	(12.91)	(9.08)		(18.71)	NVLD versus control; *p* = .841
Performance IQ	*M* = 106.5	*M* = 84	*p* = .001	*M* = 116.31	SLI versus control; *p* = .203
	(9.69)	(5.95)		(20.60)	NVLD versus control; *p* < .001

Clitic repetition (*z*-score)	*M* = .034	*M* = .966	*p* = .006	*M* = 1.05	SLI versus control; *p* = .002
	(1.1)	(.31)		(.17)	NVLD versus control; *p* = .944
Vocabulary subtest	*M* = 6.75	*M* = 11.55	*p* = .001	*M* = 10.23	SLI versus control; *p* = .015
	(2.3)	(3.01)		(3.34)	NVLD versus control; *p* = .524
Block design subtest	*M* = 10.5	*M* = 6.36	*p* = .001	*M* = 12.54	SLI versus control; *p* = .106
	(1.68)	(1.86)		(3.28)	NVLD versus control; *p* < .001
Object assembly subtest	*M* = 9.92	*M* = 6.36	*p* = .011	*M* = 10.85	SLI versus control; *p* = .678
	(3.2)	(2.0)		(2.82)	NVLD versus control; *p* = .001

**Table 3:** Characteristics of participants included in the SPM analysis. Standard deviations in parentheses.

	SLI	NVLD	*t*-test	CNTR	*t*-test
*N*	7	7		7	

Male	6	6		6	

Chronological age (yrs)	11.38 (2.9)	11.02 (2.66)	*p* = .72	11.7 (3.21)	All *p*s > .59

Full scale IQ	99.57 (10.28)	95.38 (7.62)	*p* = .60	118.14 (14.35)	SLI versus control; *p* = .16 NVDL versus control; *p* = .004

Verbal IQ	85.29 (9.41)	106.75 (8.92)	*p* < .001	118.71 (14.5)	SLI versus control; *p* < .001 NVDL versus control; *p* = .11

Performance IQ	110.43 (9.27)	84.5 (4.68)	*p* < .001	113.57 (15.59)	SLI versus control; *p* = .65 NVDL versus control; *p* < .001
