# Supplementary material for: Processing Sentences with Literal versus Figurative Use of Verbs: An ERP Study with Children with Language Impairments, Nonverbal Impairments, and Typical Development
Source: Behav Neurol. 2015 Jul 12;2015:475271. doi: 10.1155/2015/475271 (PMC4515291; doi:10.1155/2015/475271)
Supplement: Supplementary file 1 — The table contains a list of all verbs used in the verb-object combinations, and (in the corresponding columns to the right) of the four objects that were combined with each of the verbs, subdivided into concrete and abstract. The upper noun in each cell represents the congruent object, whereas the lower noun represents the incongruent object, for each of the two subgroups. Verbs were used in their infinitive form, and objects were introduced by a determiner (either a definite or indefinite article), chosen so as to preserve grammatical plausibility and accuracy with respect to Italian rules and use. [file 475271.f1.pdf]

List of experimental stimuli - each verb listed next to its abstract (congruent, incongruent) and concrete (congruent, incongruent) objects.

VERBS	ABSTRACT OBJECTS	CONCRETE OBJECTS
creare (create)	speranza (hope)<br>pazienza (patience)	statua (statue)<br>sasso (stone)
preparare (prepare)	lezione (lesson)<br>speranza (hope)	frittata (omelette)<br>pioggia (rain)
spegnere (estinguish)	entusiasmo (enthusiasm)<br>ordine (order)	computer (computer)<br>esercizio (exercise)
fare (do)	riposino (nap)<br>mente (mind)	spremuta (juice)<br>polso (wrist)
scaricare (discharge)	rabbia (anger)<br>speranza (hope)	camion (lorry)<br>insetto (insect)
avviare (start)	discorso (discourse)<br>salute (health)	motore (engine)<br>limone (lemon)
rompere (break)	silenzio (silence)<br>passion (passion)	polso (wrist)<br>bimbo (baby)
accendere (light)	speranza (hope)<br>esempio (example)	televisione (television)<br>borsa (bag)
curare (cure)	salute (health)<br>scossa (shock)	bimbo (baby)<br>dentifricio (tooth-paste)
completare (complete)	programma (programme)<br>amicizia (friendship)	palazzo (building)<br>matita (pencil)
tirare (pull)	somma (sum)<br>lezione (lesson)	sasso (stone)<br>sole (sun)
eseguire (perform)	ordine (order)<br>attenzione (attention)	esercizio (exercise)<br>camion (lorry)
seguire (follow)	istinto (istinct)<br>somme (sum)	strada (street)<br>camicia (shirt)
spezzare (break up)	legame (tie)<br>rabbia (anger)	matita (pencil)<br>strada (street)
portare (bring)	esempio (example)<br>riposino (nap)	borsa (bag)<br>casa (house)
catturare (capture)	attenzione (attention)	insetto (insect)

	relazione (relation)	vestito (dress)
spremere (squeeze)	mente (mind)<br>progetto (project)	limone (lemon)<br>lettera (letter)
rubare (steal)	parole (words)<br>legame (tie)	palla (ball)<br>muro (wall)
raccogliere (pick up)	forza (strength)<br>noia (boredom)	fiori (flowers)<br>palazzo (palace)
coltivare (cultivate)	passione (passion)<br>parole (words)	pomodori (tomato)<br>computer (computer)
scrivere (write)	rima (rhyme)<br>silenzio (silence)	lettera (letter)<br>pomodori (tomatoes)
ascoltare (listen)	lamento (complaint)<br>forze (strength)	pioggia (rain)<br>fiori (flowers)
stendere (lay down)	progetto (project)<br>bisogno (need)	vestito (dress)<br>motore (engine)
finire (finish)	pazienza (patience)<br>istinto (istinct)	dentifricio (tooth-paste)<br>palla (ball)
superare (overcome)	ostacolo (obstacle)<br>rima (rhyme)	fiume (river)<br>frittata (omelette)
allacciare (fasten)	amicizia (friendship)<br>programma (programme)	camicia (shirt)<br>spremuta (juice)
chiudere (close)	relazione (relationship)<br>entusiasmo (enthusiasm)	casa (home)<br>statua (statue)
prendere (take)	scossa (shock)<br>lament (lament)	sole (sun)<br>fiume (river)
soddisfare (satisfy)	bisogno (need)<br>ostacolo (obstacle)	bambino (child)<br>televisione (television)
abbattere (fell)	noia (boredom)<br>discorso (speech)	muro (wall)<br>bambino (child)
